# Deep Convolutional Neural Network-Based Visual Stimuli Classification Using Electroencephalography Signals of Healthy and Alzheimer’s Disease Subjects

**DOI:** 10.3390/life12030374

**Published:** 2022-03-04

**Authors:** Dovilė Komolovaitė, Rytis Maskeliūnas, Robertas Damaševičius

**Affiliations:** 1 Department of Multimedia Engineering, Kaunas University of Technology, 51368 Kaunas, Lithuania; dovile.komolovaite@ktu.edu; 2 Department of Applied Informatics, Vytautas Magnus University, 44404 Kaunas, Lithuania; robertas.damasevicius@vdu.lt

**Keywords:** Alzheimer’s disease, electroencephalogram, SSVEP, visual stimuli classification, face inversion, generative adversarial networks, data augmentation, deep learning

## Abstract

Visual perception is an important part of human life. In the context of facial recognition, it allows us to distinguish between emotions and important facial features that distinguish one person from another. However, subjects suffering from memory loss face significant facial processing problems. If the perception of facial features is affected by memory impairment, then it is possible to classify visual stimuli using brain activity data from the visual processing regions of the brain. This study differentiates the aspects of familiarity and emotion by the inversion effect of the face and uses convolutional neural network (CNN) models (EEGNet, EEGNet SSVEP (steady-state visual evoked potentials), and DeepConvNet) to learn discriminative features from raw electroencephalography (EEG) signals. Due to the limited number of available EEG data samples, Generative Adversarial Networks (GAN) and Variational Autoencoders (VAE) are introduced to generate synthetic EEG signals. The generated data are used to pretrain the models, and the learned weights are initialized to train them on the real EEG data. We investigate minor facial characteristics in brain signals and the ability of deep CNN models to learn them. The effect of face inversion was studied, and it was observed that the N170 component has a considerable and sustained delay. As a result, emotional and familiarity stimuli were divided into two categories based on the posture of the face. The categories of upright and inverted stimuli have the smallest incidences of confusion. The model’s ability to learn the face-inversion effect is demonstrated once more.

## 1. Introduction

Visual perception helps people understand their surroundings. However, with age, visual processing becomes more challenging. Although Alzheimer’s disease (AD) usually starts with memory impairment, it is also thought to affect vision processes. Patients with AD have been found to often experience reading difficulties and spatial disorientation [1]. This becomes even more difficult when they cannot recognize familiar faces or even themselves [2]. Because deficiencies in both memory and gnostic processes can be a consequence of AD, it is possible to determine which area is not so severely affected and whether targeted rehabilitation or other supportive applications would be possible.

**Figure 1:**
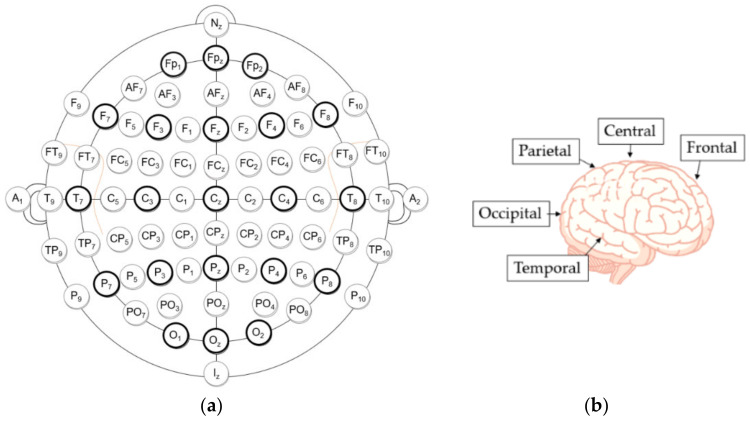
(**a**) The 10-10 electrode placement system. (**b**) Cerebral lobes and central area [14]. Label “z” indicates midline area. Odd numbers—the left hemisphere; even numbers—the right hemisphere.

Brain–computer interface (BCI) systems are known to help people with motor disabilities. They have even been adapted for faster image annotation in computer vision tasks [3]. Without any physical activity in humans, only by understanding the intentions of a person using brain signals, the system converts them into actions or commands [4,5]. BCI is generally based on the analysis of noninvasive electroencephalography (EEG) signals [6]. Due to the low cost [6] and high temporal resolution [7], EEG has been an area of interest for many researchers, such as the evaluation of psychological status [8], sports informatics [9], and biometrics [10,11]. A device consisting of electrodes measures the electrical potential of the scalp [12]. It can show brain activity in certain regions that are responsible for various tasks, such as (Figure 1):

- Frontal lobe (F)—responsible for language, problem solving, decision making, and memory [12].
- Temporal (T) lobe—mainly responsible for auditory information and verbal memory of information, as well as certain aspects of visual perception [12].
- Parietal (P) lobe—responsible for sensory information interpretation [12], texture, weight, and object recognition [13].
- Occipital (O) lobe—responsible for early visual perception [12].

Many studies suggest that EEG can accurately predict some brain diseases, such as epilepsy [15], Alzheimer’s disease, and autism [16]. However, the analysis of EEG signals is a very difficult task because they are high dimensional and nonstationary [6] and have a poor signal-to-noise ratio [7,14]. Most importantly, experiments with EEG signals usually have relatively few data, and brain impulses are specific to each user, so BCI systems must be calibrated [17]. Despite the shortcomings of EEG, there is a potential for BCI systems to be adapted for patients with memory impairment: to provide the necessary information about visual stimuli, the relationship status with a visible person, and more.

Different stimuli cause different brain reactions. The path of visual perception begins in the retina and extends to the occipital lobe [16]. From the early visual cortex, visual processing spreads to the lateral occipital lobe during the first few milliseconds (about 200 ms) [18]. Electrical activity causing visual perception is observed throughout the pathway with different response patterns depending on the visual stimulus [14]. Signal changes can occur in different channels at different times and with different amplitudes. Neural oscillations evoked by stimuli constitute event-related potential (ERP). If the stimulus presented is visual, it may also be referred to as visual event-related potential (VEP) [19]. Additionally, if the ERP is averaged over the same stimuli, then it is called the Grand Average ERP [19] and is analyzed by peak amplitude (P—positive; N—negative) and the timing of the peak in milliseconds [20].

The visual processing of a human face is very complex. One can glean information such as a person’s identity and emotional state from faces [18]. The face-inversion effect is noticeable more quickly than emotions, as it flips the entire face instead of focusing on facial features. The most reliable facial marker in EEG studies is the N170 component, a face-sensitive ERP component with a negative wave amplitude deflection that occurs around 170 ms after the presentation of a face [21]. In this component, the face-inversion effect (FIE) has a significant and consistent delay (about 10 ms) [22], as has been demonstrated in many studies [23]. Only after the position of the face is defined does the brain analyze features such as emotions. The emotional effect in the EEG signal occurs 310–1000 ms after the stimulus onset. On the other hand, the N250 component is more prominent in familiar faces than in unknown faces. N250 has been shown to increase when a face becomes more familiar in terms of face individuation [24]. Inverted face information was also found to take longer to encode compared to an upright face [17]. Meanwhile, a study analyzing emotional expressions found that fearful faces caused significantly higher and longer-lasting negative activity in the signal compared to neutral faces [25]. This emotional effect begins approximately 316 ms from the stimulus onset and remains for another 684 ms [25]. According to [18], the temporal stimulus encoding was divided into three phases: 1. configuration (140–160 ms); 2. face individualization (180–300 ms); and 3. changeable aspects of faces such as emotion (310–1000 ms). In addition, other studies also found that the sensitivity for familiar faces is present at about 250 ms [18], and the 320–480 ms time window achieves the best performance for visual stimulus classification [23]. This suggests that the key areas of the brain for visual classification are related to higher-level cognitive processes rather than visual processing only.

In recent years, deep neural networks (DNNs) have received increasing attention from researchers for a variety of classification tasks by using EEG data: alcoholism detection [26], predicting early stages of schizophrenia [27], classifying motor imagery to assist brain–computer interfaces [6,28], determining the stage of AD [29], and even the stages of visual processing [30]. The growing interest in visual perception may open up more opportunities to adapt BCI systems to visually impaired people [30]. However, because small training sets are usually available for BCI design, shallow networks are the only ones that have been identified as useful and promising [17]. They have fewer parameters and fewer hidden layers; therefore, they are not as prone to overfitting as the richer deep models [13,31]. Deep learning methods have also been shown to reduce the need for feature engineering, as the use of raw EEG data achieves impressive results [15,32]. Finally, we have a problem in that artefacts in EEG present a difficulty in BCI, as it is often used to decipher motor preparation and imagination. To combat this, Mammone et al. suggest using maps embedded in a volume and using this as input to a deep convolutional neural network CNN [33].

Artificial reconstruction with high sampling rates and sensitivity is difficult due to the nature of EEG signals. The link between EEG data associated with emotions, a coarse label, and a facial expression image was established in the study [34] using a conditional generative adversarial network (cGAN). The authors of [35] recommend using a Generative Adversarial Network with Wasserstein Distance and Temporal-Spatial-Frequency Loss to reconstruct EEG signals. Luo et al. developed a Conditional Wasserstein GAN (CWGAN) framework for EEG data augmentation to improve EEG-based emotion recognition in order to overcome the shortage of data when assessing emotions [36]. Bhat et al. suggest adding nine descriptive features extracted from the original data to the GAN implementation [37]. A key goal in the related Alzheimer’s research is to determine how it affects the ability to process contextual information and regulate threat responses, addressing the fact that structural and physiological changes in the prefrontal cortex and medial temporal lobe determine cognitive changes in advanced aging, which can eventually lead to the patterns of cognitive dysfunctions seen in patients with AD/MCI [38] with a very complex pathophysiological basis, dependent on different biomarkers affecting the cognitive decline [39]. The classification of visual information is a daily human function; however, can we automatically identify visual stimuli based on brain signals? Additionally, how does automatic classification respond to Alzheimer’s patient data?

The purpose of this study is to investigate whether the visual stimuli of a patient with AD can be detected in the same way as in a control (healthy) group. We also want to determine whether a trained CNN can learn the key components that encode facial-related information, even though some areas of the brain of the AD patient may be damaged. However, assuming that memory rather than visual processes is the consequence of the disease, the model is expected to understand visual perception in a very similar way. The task of classifying visual stimuli is challenging even with data from a group of healthy young people, but this study covers the older group. To the best of our knowledge, such a study to examine the visual perception of the elderly, including a patient with AD, has not been done before.

The following objectives were set accordingly:

1. Investigate whether a trained CNN model can detect categories related to emotions and facial inversion: fear/upright, fear/upside-down, neutral/upright, and neutral/upside-down.
2. Investigate whether a trained CNN model can detect categories related to familiarity and facial inversion: famous/upright, famous/upside-down, unfamous/upright, and unfamous/upside-down.
3. Investigate how pretrained model weights with augmented data affect model performance.

The structure of the paper is as follows: Section 2 describes the classification tasks for visual stimuli performed for different stimuli. In Section 3, we provide information on classification methods using convolutional neural networks. Section 4 presents an experimental design with information about participants and data. Section 5 provides detailed information on preprocessing, training, improvements to the original model, and evaluation. Finally, we present the discussion and summary results in Section 6.

## 2. Related Work

In recent years, the challenge of visual classification has been increasingly addressed. In the visual context, the most popular visual stimuli are movement imagery data because they can be directly applied to individuals with motor disorders in BCI systems [38,39,40,41,42]. However, the application of visual stimuli can also be applied in many areas: to perform faster image annotation tasks [3], to understand the processes of visual perception in the brain, and to help those with visual impairments. The medio-frontal negativity, a component of the event-related brain potential generated in the ACC/mPFC, tracks the timing of salient events and reports an error signal when the aversive outcome is delayed or predicted from an expected time, according to the study [40].

Because visual encoding is a complex task, many studies rely on the classification of binary data [43,44,45]. Even then, performance is not always excellent, as magnetoencephalography (MEG) recordings provide 64% accuracy in predicting the face compared to a scrambled face [46], and another study using EEG data has less than 65% accuracy in detecting upright versus inverse facial stimuli [47]. Nevertheless, the effect of facial inversion has been extensively studied, showing that the face-sensitive N170 component is higher in amplitude for faces [48] and the prediction of facial inversion increases from 125 ms to 375 ms after stimulus onset [47]. However, there is evidence that N170 is also sensitive to differences in facial category: identity and emotion [48]. In terms of emotion prediction, more research has been carried out to determine the emotion that a subject experiences when seeing the various images presented [49,50]. Unfortunately, no studies have attempted to predict a person’s ability to correctly identify other person’s emotions, although the effect of emotion stimuli has been observed in the past [25]. In addition, research on familiarity aspects has shown that the successful acquisition of long-term memory information is necessary to recognize a familiar face [51]. The visual memorability of the media content was examined to determine how easily the image could be memorized [52]. Research related to memory processes is important in advertising, education, treatment of memory-related diseases, and other areas. The related work is summarized in Table 1. The highest accuracy in predicting visual stimuli, approximately 83%, was achieved with the RNN model in a study with 40 classes of visual stimuli, but the model was developed using both image data and EEG signals. Therefore, if a single data source is not available, a classification model cannot be used [53]. Another study, also using an RNN model, which can capture long-term dependencies over time, achieved an accuracy of 61.74%, while DeepConvNet achieved 64.82% [5]. The authors of the article state that this is due to the complex structure of DeepConvNet and the larger number of parameters [5].

## 3. Methods

### 3.1. Raw EEG Classification Methods

The EEGNet, DeepConvNet and EEGNet SSVEP deep neural network models have been shown to be effective and useful in a variety of classification tasks [54]. Further, EEGNet SSVEP is specifically designed to classify visual potential signals [31]. Therefore, it is valuable to start analyzing EEG data with these methods to obtain benchmark results.

#### 3.1.1. EEGNet Architecture

EEGNet is a compact CNN than can be used for a variety of EEG signal classification tasks, including event-related potentials (ERP). The EEGNet model can effectively extract different types of properties from the signal data [54]. The performance of cross-subject classification for ERP data is as good as using the DeepConvNet architecture. An architecture structure consists of only three convolutional layers: 2D temporal convolution, depth-wise convolution and pointwise convolution [54]. All layers use a nonlinear activation function—the exponential linear unit (ELU). The model’s input is raw EEG data, including the number of channels and time samples. The detailed structure is shown in Table 2.

The original paper experimented with the EEG data taken at 127 Hz with initial parameters such as: $F_{1}=8$, $F_{2}=16$, D=2 [54].

To limit the number of trainable parameters, the EEGNet architectural structure employs depth-wise and separable convolutions. The initial combination of 2D convolution and depth-wise convolution allows each temporal filter to learn spatial filters [55]. Meanwhile, the number of spatial filters learned from each feature map is controlled by a depth parameter [56]. After each convolution, batch normalization is performed to achieve model stability. Additionally, dropout layers are used to significantly reduce overfitting [26]. The final multi-class classification layer uses the SoftMax function [57].

#### 3.1.2. DeepConvNet Architecture

The DeepConvNet architecture is designed to be general purpose. It consists of five convolutional layers: 2D temporal convolution with an increasing number of filters and ELU activation functions, and the last layer is a dense Softmax classification. This method is better than the standard filter bank Common Spatial Models (FBCSP) algorithm, but its main advantage is that the features do not have to be predefined and the method can be applied to general cases. Additionally, DeepConvNet can learn to use spectral power modulations in different frequency bands. The length of temporal convolution should be (1, 10) for data sampled at 250 Hz [50]. The architectural structure is shown in Table 3.

#### 3.1.3. EEGNet SSVEP Architecture

The EEGNet SSVEP architecture utilizes Compact-CNN to be specifically adapted for steady-state visual evoked potentials (SSVEPs). These are events only from the visual cortex electrodes (parietal and occipital) when the visual stimulus is observed. This approach can be used to train even small data sets. The main difference between EEGNet and EEGNet SSVEP is that the kernel length of the first convolutional layer is equal to the time samples (instead of the half of the time samples), and no maximum norm weight limit is used for the final dense layer [31].

The EEGNet SSVEP network also distinguishes between phase and amplitude features. As previously stated, the first convolutional layer simulates a bandpass frequency filter by performing a temporal convolution. In the meantime, the depth-wise spatial convolutions are supposed to operate as spatial filters, reducing the data’s dimensionality. Additionally, the ELU activation function is also employed because it has been shown to perform better for EEG classification. The authors used the following initial parameters: dropout rate = 0.5, $F_{1}=96$, $F_{2}=96$, and D = 1 [58].

### 3.2. Artificial EEG Data Generation Using the VAE

The size of the training data set has a direct impact on the performance of the deep learning model [59]. The classifier may be overfitting or have poor generalization skills in the absence of reliable data. However, since large-scale EEG datasets are difficult to obtain in real life, artificial data augmentation methods have been proposed to address this issue [60].

In this paper, Generative Adversarial Networks (GAN) and Variational Autoencoders (VAE) were used. It was discovered that training a classifier using a pretrained model on synthetic data, rather than mixing actual and generated data, resulted in the biggest increase in classification accuracy. The accuracy of generalization ability achieved by training the classifier in one subject and testing it in another using VAE data improved by up to 37% [57].

**Figure 2:**
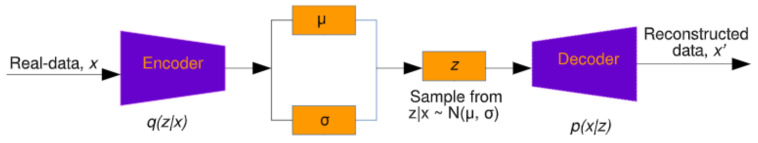
The Variational Autoencoder for generating reconstructed data [47].

VAE is an improved variant of the Autoencoder (AE) [60]. VAE differs in that it assures that the encoder output has a certain learnt data distribution [59]. A Convolutional Variational Autoencoder built from 1D convolutions can be utilized to produce synthetic EEG data (see Figure 2) [57]. Here, the input data are compressed by the encoder, and the data with important features are restored by the decoder [59].

The VAE loss function

(1) $$L=E_{q(z|x)}\left[\log\left(p\left(x|z\right)\right)\right]-KL(q(z|x)||p\left(z\right))$$

The encoder is trained to learn the mean μ and variance σ of the latent space. From here, the vector z is sampled using a Gaussian distribution $z=N\left(\mu ,\sigma \right)$. The decoder is then trained to recreate a realistic output. The custom loss function is defined in Equation (1), where KL is the Kullback–Leibler distance.

## 4. Materials

### 4.1. Participants and Data Source

The EEG data were taken from the Figshare website and are publicly available [61]. The experiments were conducted in 2017–2018 and involved a total of nine women, including one patient with AD. All women were between the ages of 63 and 70. One patient was a 67-year-old right-handed woman with facial recognition problems identified by the initials “MCG” [2]. Although the dataset has few subjects, the statistical analysis performed in [2] using the repeated measures ANOVA test and nonparametric bootstrapping showed statistically significant differences between the EEG data (N170, N250 and N400 components) of the healthy participants and the AD subject at a statistical threshold of 0.05.

There was a total of 576 stimuli trials per subject. After the artifacted epochs were removed, approximately 477 trials remained for each of the control subjects, 467 trials for the oldest subject, and 426 trials for an Alzheimer’s patient. Therefore, a total of 4234 EEG signal segments were used in experiment no. 2. Accordingly, for experiment no. 3 around 567 trials were employed for each of the control subjects, 554 trials for the oldest subject, and 430 trials for Alzheimer’s disease patient. Consequently, a total of 4955 data segments were used. For control subjects, the overall experiment duration ranged from 34 to 43 min (Experiment 2) and from 38 to 49 min (Experiment 3). Meanwhile, for an Alzheimer’s patient, trials lasted 88 min (Experiment 2) and 94 min (Experiment 3). Note that the numbering of experiments follows the numbering scheme set in [61].

### 4.2. Experiment Design

The minimizing of noise, such as head and eye movements, was ensured in all experiments. In three separate trials, different stimuli were investigated. Participants in experiments 2 and 3 (the numbering according to the dataset reported in [2] was used) had to determine whether the stimulus was upright or inverted. The effect of facial emotion (neutral or fearful expression) was studied in experiment 2. Additionally, experiment 3 looked at the influence of familiarity: whether the faces were famous or not. All studies were carried out on different days for the Alzheimer’s patient [2]. Examples of visual presentations are given in Table 4. There is also another important detail in that the images in the second experiment are in color, and the images in the third are grey.

The visual stimulus was presented for 300 ms. After a 1000 ms pause, subjects pressed two different keys to identify face position, and then the next trial was presented. The EEG electrode positions were composed according to the 10-10 international system, and four additional electrodes were also used to monitor blinking and eye movements [2]. The data provided consist of 64 electrodes and signals with a sampling frequency of 250 Hz.

### 4.3. User Responses

**Figure 3:**
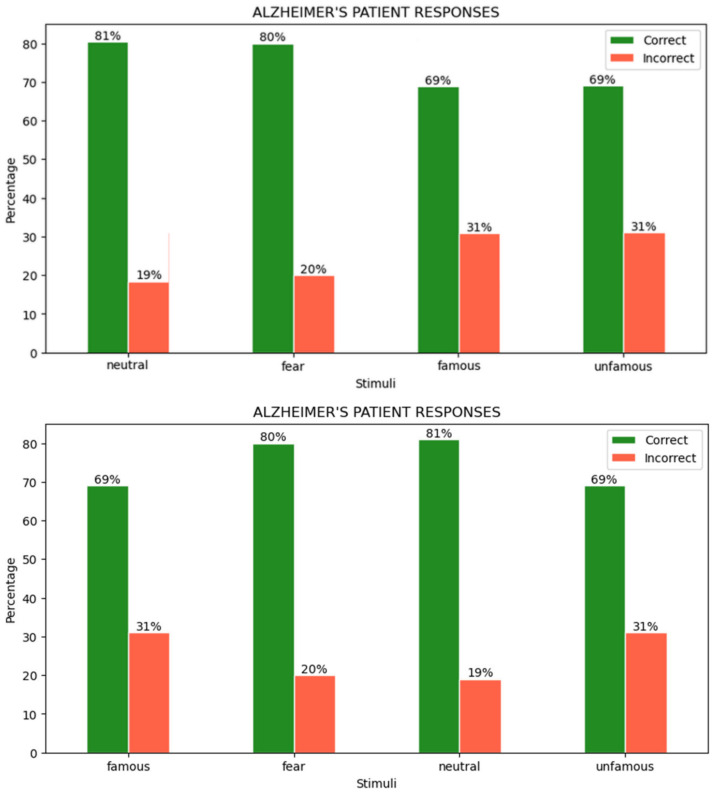
Responses of the AD patient to face inversion in different visual stimuli.

During the experiments, all participants were asked to differentiate between facial position (inverted or upright) according to different color scales, and emotional and familiarity aspects. By analyzing the experimental data, the percentages of correct and incorrect responses in the AD patient were calculated (see Figure 3).

A patient with AD detects the facial position poorly in the familiarity photos provided, with a total of 31% inaccurate responses. However, since the photos are black and white, the properties of the dark hair merge in with the environment and this can cause more difficulties for an AD patient. Meanwhile, with an average of 20% incorrect answers, the recognition of face position by presenting stimuli in colorful images with different facial emotions is slightly better. Looking at Figure 3 alone, familiarity or emotion aspects do not appear to have a strong influence on the responses of MCG patients. The distribution of correct answers between these categories is almost the same. However, color can have a greater effect.

## 5. Experiment

**Figure 4:**
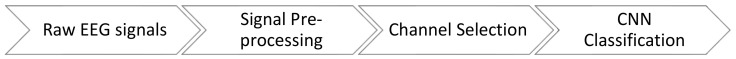
Framework of the experimental approach.

The experiment consists of the following steps (Figure 4), which are explained in more detail in the following subsections.

### 5.1. EEG Data Preprocessing

Raw multichannel EEG data were first filtered using an FIR bandpass filter using the 4 Hz and 40 Hz limits. Theta waves (4–8 Hz) and alpha waves (8–12 Hz) have been shown to be the most active in visual tasks [62]. However, beta (15–31 Hz) and gamma (32–70 Hz) bands provide information about cognitive processes related to visual perception [19]. Many studies use different frequency windows: 14–71 Hz [19], 9–30 Hz [41], 12–65 Hz [5], 1–40 Hz [2], and 4–40 Hz [4]. The frequency filtering step also helps prevent physical artifacts such as head movement, blinking, or device-to-head connectivity problems [12].

**Figure 5:**
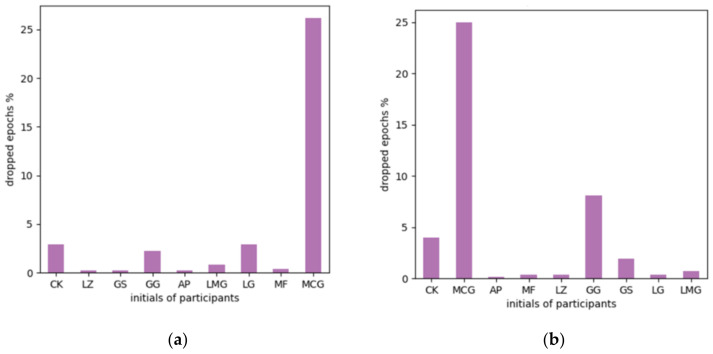
Rejected epochs due to artifacts. The percentage of dropped epochs for each participant: (**a**) Eexperiment No. 1 and (**b**) Eexperiment No. 2.

Then, epochs were extracted with a time window from 200 ms before the stimulus to 800 ms after, and the baseline correction was applied using the prestimulus interval. In this way, 1 s-long epochs remain around the induced visual stimulus, which includes early and late image processing features. Epochs were rejected if the peak-to-peak signal value was greater than 150 μV (Figure 5). Finally, data were normalized between −1 and 1 due to the deep learning sensitivity to scaling, as mentioned in [63].

### 5.2. Channel Selection/Selection of Electrodes

**Figure 6:**
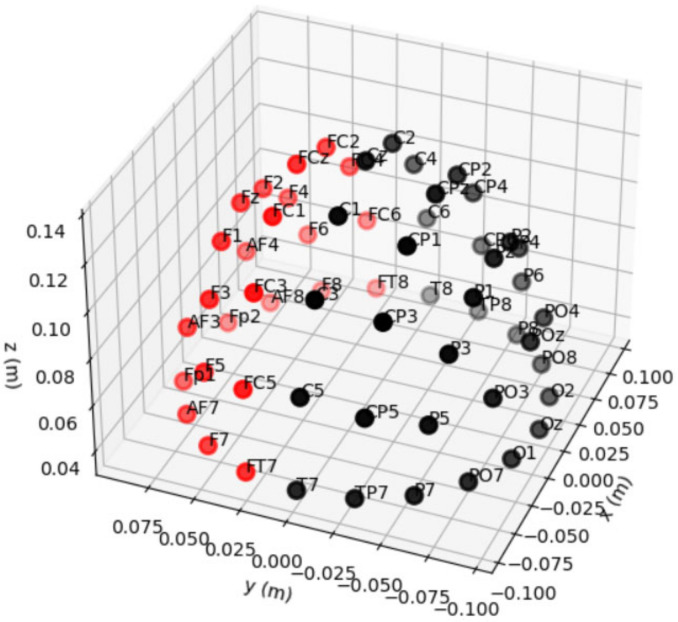
Sensor positions. Selected channels are black and bad channels are red.

Reducing the number of channels can lead to better accuracy. Using the knowledge of previous studies, we know that the most important areas of the brain for image classification are those outside the visual cortex [13,53]. It was discovered that the use of channels from the frontal cortex or the somatosensory cortex alone or in combination (Fp, F, T, C) greatly reduced the quality of recognition [64]. Although some sources claim that channels F, Fp, and FC give better accuracy [13,53], these areas are adjacent to the eye and can capture different eye movements in response to various visual stimuli. Because the visual cortex (O) and recognition cortex (P) channels improve visual stimuli prediction accuracy but are not the only significant areas, a larger spectrum of channels is required [13,53]. For example, channels in the temporal (T) lobe may also contain information regarding visual perception [12]. This channel selection chooses all channels with the letters O, PO, P, TP, T, CP, and C. Out of a total of 64 channels, this selection left 35 channels (see Figure 6).

### 5.3. Data Augmentation Using VAE

The Variational Autoencoder model was trained using EEG signals. The VAE encoder was developed using 2D convolution, the LeakyReLU activation function and batch normalization. The dimensions were then flattened, and dense layers were employed to obtain the mean and standard deviation of the Gaussian distribution. These statistic metrics were used to calculate the loss function. The aim was to train the NN to gain the knowledge of the VAE about influential features. In this case, the network weights are not initiated randomly, and have more information about possible data distributions. According to sources, the use of synthetic data improves the performance of the classifier, as it also performs the noise removal function, as the study [57] demonstrated that training with synthetic data improves model performance by up to 16% and removes subject-specific features from EEG signals, making the calibration step unnecessary.

In the next step, the decoder took the encoder’s output and replicated the same size output as the encoder input using a dense layer, deconvolution, and batch normalization. In Table 5, the predefined model hyperparameters are listed. Each sample with 32 channels and 250 time points passed through the encoder and decoder while the model learned to reduce the loss function. Training was carried out independently for each class, with a validation and test ratio of 20%. Table 6 lists the performance metrics.

**Figure 7:**
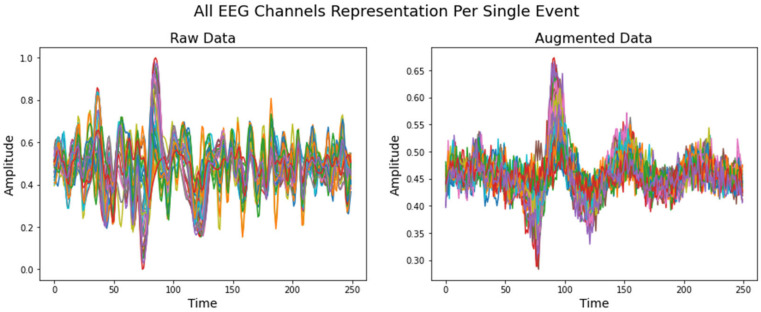
Raw EEG signal from validation dataset compared to artificially created data. The sample was taken from a model that had been trained using famous/upright class inputs.

**Figure 8:**
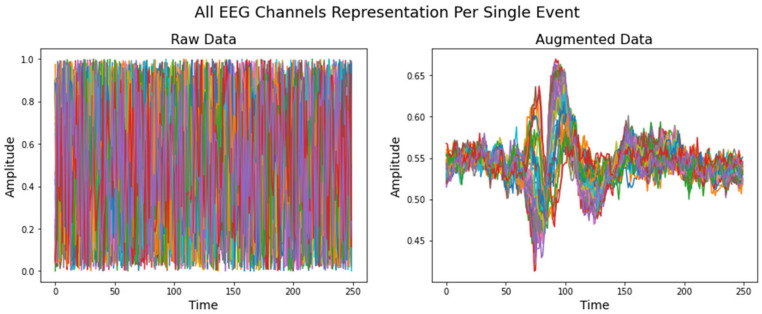
Randomly generated data compared to synthetic augmented data. The sample was taken from a model that had been trained using famous/upright class inputs.

Randomly generated data from 0 to 1 value were provided to the model when the VAE learned to replicate the input distribution. The VAE model recreated the pattern for each individual class, resulting in 500 samples for each. Figure 7 and Figure 8 demonstrate an example of how the VAE model works for a single event from the validation dataset and a randomly generated dataset, respectively. *Y* axis is in microvolts normalized from 0 to 1; *X* axis displays time points in 1 s intervals, sampled at 250 Hz.

The developed synthetic data samples are used in the pretraining stage because they have been shown to give better results than mixing augmented and raw data. The pretraining section is described later in the study.

### 5.4. Training Setup

Python programming language was used with tensorflow-gpu version 2.4 (Google Brain, Mountain View, CA, USA). Models were trained on an NVIDIA-SMI 495.44 GPU with CUDA (Compute Unified Device Architecture) version 11.2 (NVidia Corporation, Santa Clara, CA, USA).

**Figure 9:**
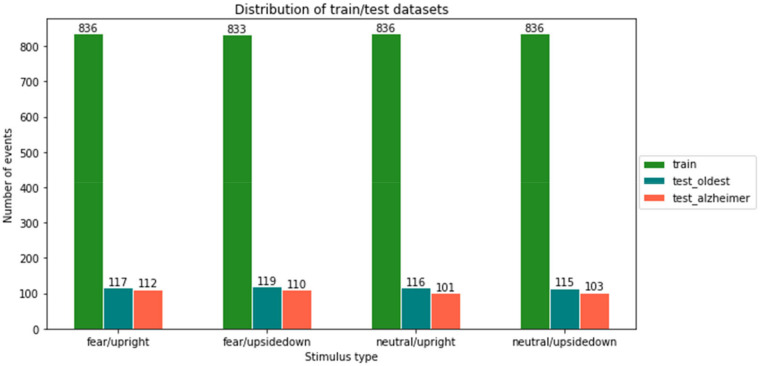
Distribution of training and testing samples for emotion- and view-type stimuli.

**Figure 10:**
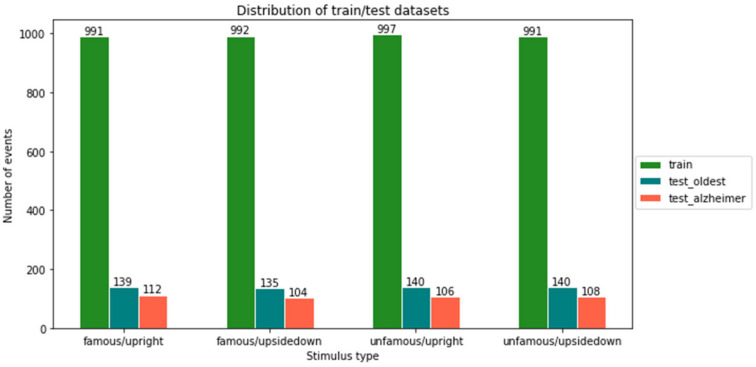
Distribution of training and testing samples for familiarity- and view-type stimuli.

Classification methods were trained on control data and tested on the AD patient data. However, one control subject (the oldest) was left out of the training data set to confirm the predictor of a healthy person’s stimulus using the unseen individual brain signals. In this way, the possibilities of a user-independent model were tested. For training and validation, 75%/25% ratios were used to split the data. As seen in Figure 9 and Figure 10, class imbalance is not present in any of the experiments.

Labels for the visual stimulus class were one-hot-encoded. The model was trained for 500 iterations using the Adam [65] optimizer, with a batch size of 64 samples. We observed that the ability of the model to generalize deteriorates with larger batch sizes [66]. Early stop monitoring was used, so if loss begins to increase in the test dataset, training is stopped to prevent overfitting. The values of the training hyperparameters are shown in Table 7.

### 5.5. EEGNet SSVEP Model with Regularization

In general, the architecture of an EEG data model architecture should not be complicated, as the number of training samples is often limited [5]. This is extremely important to prevent overfitting and to maximize generalizability. To improve the best-performing model EEGNet SSVEP, the L1 and L2 regularization methods were added to the final fully connected layer with both regularization penalty values equal to 0.001. The addition of constraints to the model weights has been shown to minimize the complexity of the model [26]. One study improved the accuracy of identifying motor movements from the EEG data by 2% using the EEGNet model with regularization [56]. Consequently, tests were conducted to assess whether the addition of regularization to the model improves visual stimulus classification using a similar model [67].

### 5.6. Training

Three established models from prior research were used to train the CNN model: DeepConvNet, EEGNet, and EEGNet SSVEP, as well as a modified technique, called EEGNet SSVEP with regularization. The raw signal data were used to train all four models. The first time, the models were trained using randomly initiated weights, and the second time, the models were trained with weights that were pretrained using artificially generated data. The results were evaluated for both types of stimuli, familiarity and emotion.

#### 5.6.1. Pretraining with Augmented Data

The pretraining was conducted with 500 artificially generated samples per class from the VAE model. The data were divided into training and testing datasets in a 70%/30% ratio and shuffled. Each model was trained for 300 epochs unless the validation loss stopped improving during the final 50 epochs. The Adam optimization technique was used with a batch size of 128 and a learning rate of 0.00001. Almost all models were able to correctly classify the stimulus types with 100% accuracy (see Table 8).

These pretrained model weights were saved and later used to classify real EEG data to improve model accuracy.

#### 5.6.2. Familiarity and View Stimuli Classification

**Figure 11:**
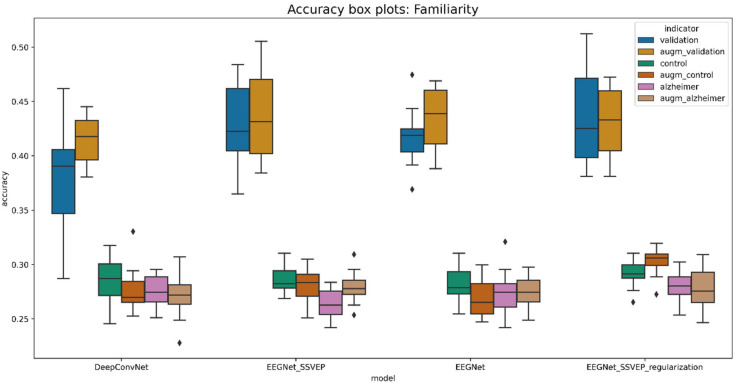
Boxplot for evaluating the accuracy of familiarity stimulus types.

**Figure 12:**
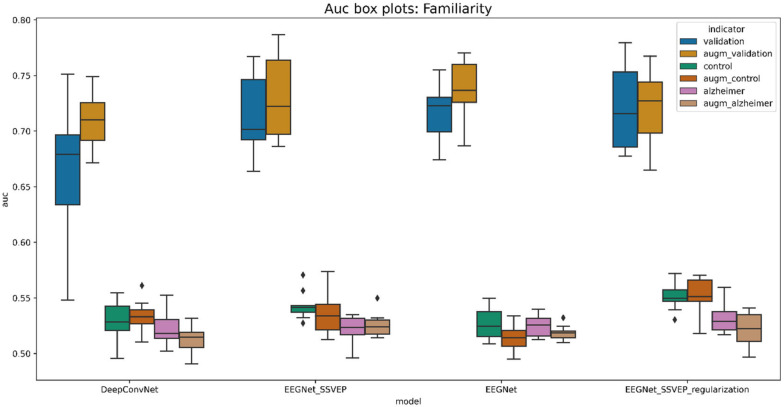
Boxplot for evaluating the AUC of familiarity stimulus types.

The evaluation was performed using a two-fold cross-validation technique 5 times, meaning that the cross-validation procedure was repeated 5 times, dividing the data into 2 folds. This generates the random data partitions and provides better insights regarding model generalization. The boxplot diagrams in Figure 11 and Figure 12 summarize the findings.

The presented boxplots show accuracy and area under the curve (AUC) metrics. Overall, accuracy is an excellent indicator for balanced classes since it reflects the percentage of correct classifications. If the accuracy is greater than 25%, then the classifier performs better than the random classifier. Meanwhile, the AUC metric indicates whether a classifier is more confident than random if it is more than 50%.

A pretrained EEGNet SSVEP with added L1 and L2 regularization is the best model for predicting familiarity stimulus types. This model has been demonstrated to be effective for visually evoked potentials. In the validation dataset, the trained model accurately identified 43.25% of the stimulus, compared to 30.23% in the control group and 27.72% in the data of Alzheimer’s patients. As a result, the model for the oldest patient is 5.23% and that for the Alzheimer’s patient is 2.72% better than the randomized one. When comparing all models trained from randomly initiated weights to pretrained weights, the difference is less significant, yet validation data performance improves by 1.5% on average. The average performance metrics are given in Table 9.

**Figure 13:**
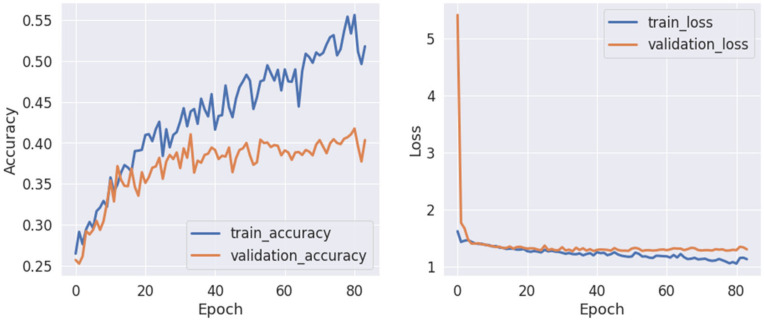
A performance history of the pretrained EEGNet SSVEP model with regularization for familiarity stimulus types.

Following the identification of the best-performing model, some research was carried out looking into one of the best training scenarios. Training was terminated after roughly 80 epochs to prevent the model from overfitting, according to the model performance history (see Figure 13).

**Figure 14:**
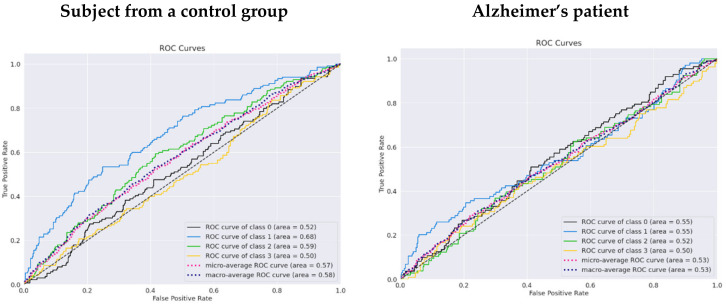
The ROC curves for a subject from a control group and a patient using the pretrained EEGNet SSVEP model with regularization trained to distinguish the familiarity stimulus types.

According to the receiver performance characteristics (ROC) curves (see Figure 14), there is evidence that the results in the unfamous/upside-down class (number 3) have the lowest performance, with an AUC of 0.5 for a control group subject and AUC of 0.5 for an Alzheimer’s patient. The highest AUC of 0.68 was reached for the class famous/upside-down and is the only class that can be classified as acceptable.

**Figure 15:**
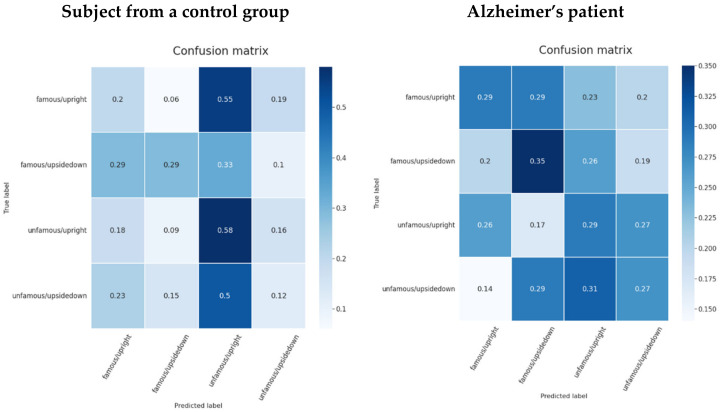
Confusion matrices for a subject from a control group and a patient using the pretrained EEGNet SSVEP model with regularization trained to distinguish the familiarity stimulus types.

In the confusion matrices (see Figure 15), the second class (unfamous/upright) is the one that is predicted the most for each true class, resulting in a high false positive rate. The famous/upside-down and famous/upright have distinguishing characteristics for a control group individual. Both classes show lower prediction rates for the opposite face position. That is, the classifier model was able to start learning the facial inversion effect, but the familiarity signal patterns were more difficult to identify. On the other hand, in Alzheimer’s patient data, unfamous/upside-down had the lowest score for the opposite class famous/upright and the same situation occurred with the unfamous/upright class when famous/upside-down had the lowest prediction score. Although the model was unsure which class would be the best fit, it was definite that the opposite class would not.

#### 5.6.3. Emotion and View Stimuli Classification

**Figure 16:**
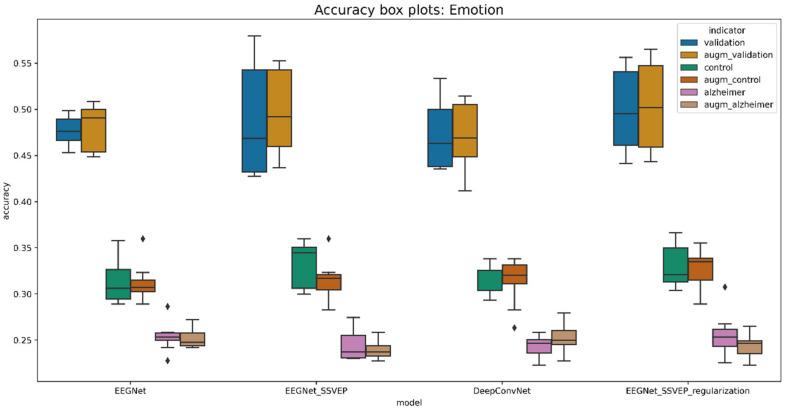
Boxplot for evaluating the accuracy of emotion stimulus types.

**Figure 17:**
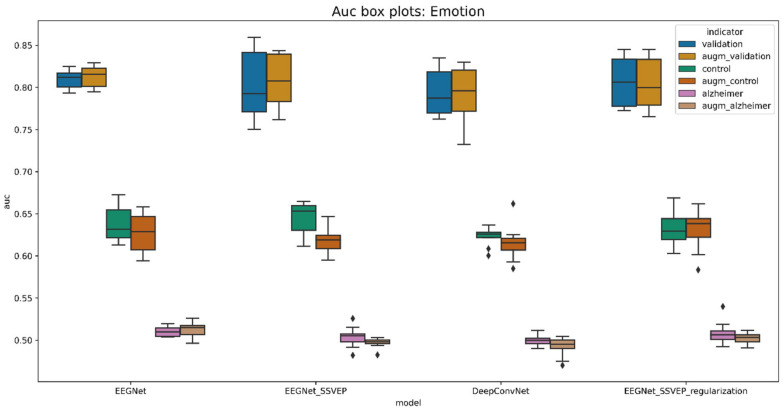
Boxplot for evaluating the AUC of emotion stimulus types.

The analysis was carried out in the same way as in the previous section. Boxplots were created using a 2-fold cross validation approach. The results are presented in Figure 16 and Figure 17.

When the differences are not significant, it is more difficult to decide which model performed best. On the other hand, the model with the best validation accuracy was the same as for the prediction of the type of familiarity stimulus. Validation data were better learned by pretrained EEGNet SSVEP with the regularization of L1 and L2. However, when it came to generalization, this model did not perform as well as the non-pre-trained model on control group subjects. In the validation dataset, the pretrained model in the augmented data had 50.2% accuracy, 32.75% accuracy in the control subject dataset, and 24.41% accuracy in the Alzheimer’s patient data. The emotion prediction for the oldest patient is 7.75% better than the random classifier, while it is 0.59% worse for an Alzheimer’s patient. Meanwhile, comparing the results of the randomly initiated weights and the pretrained pretrained weights, the weights only benefit by 0.5% on average. Table 10 shows the values of the average performance metrics.

**Figure 18:**
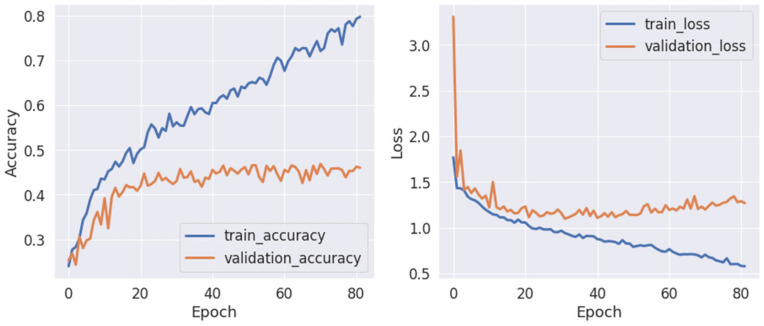
A performance history of the pretrained EEGNet SSVEP model with regularization for emotion stimulus types.

The selected best-performing model (pretrained EEGNet SSVEP with regularization) was further investigated. The model was trained for more than 80 epochs before the validation loss began to increase. The gap between training and validation was wider and the model tended to overfit, although the architecture was designed specifically to avoid this. The model performance history is shown in Figure 18.

**Figure 19:**
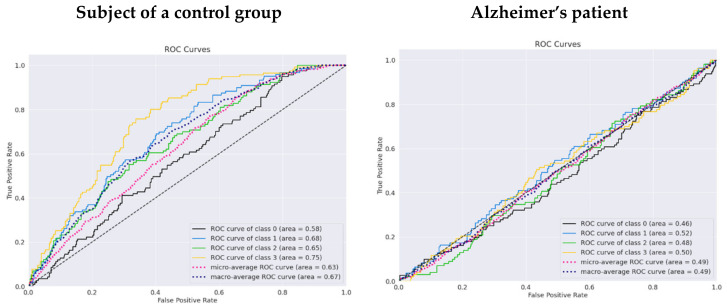
ROC curves for a subject in a control group and a patient using the pretrained EEGNet SSVEP model with regularization trained to distinguish the types of emotion stimulus.

A clear distinction from a random classifier can be seen in the ROC curve for a participant in the control group (see Figure 19). The AUC for a fear/upright class is 0.58, whereas neutral/upside-down has an AUC of 0.75. However, two classes performed worse than the random classifier for an Alzheimer’s patient. Fear/upright and neutral/upright are the two classes with the highest false positive rate.

**Figure 20:**
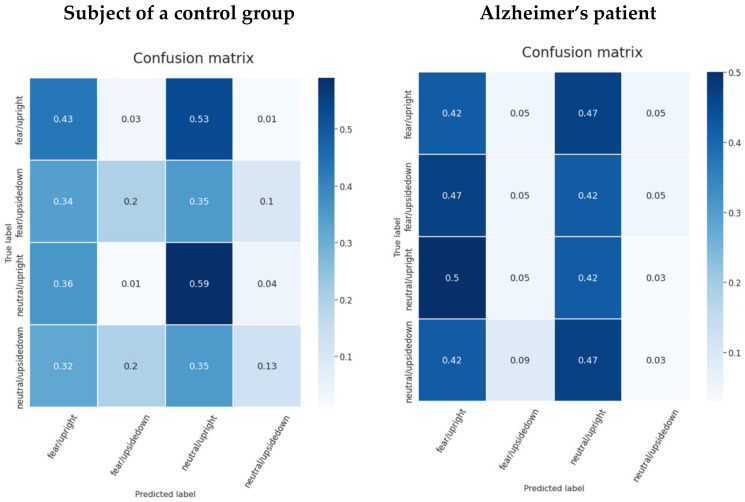
Confusion matrices for a subject from a control group and a patient using the pretrained EEGNet SSVEP model with regularization trained to distinguish the emotion stimulus types.

Figure 20 shows the pattern of model confusion for the emotion and face-inversion effect stimuli. The model is prone to wrongly classifying two groups, while overlooking the rest. This pattern may be seen in both the Alzheimer’s patient data and the control group patient data. However, two classes with the highest AUC values of 0.75 and 0.68 reveal some distinction between the face-inversion effect and yet none for the emotion type. Face inversion is a higher and more obvious information encoded in the signal than the emotion that the individual displays.

## 6. Discussion

To our knowledge, this is the first study to attempt to identify minor facial characteristics using raw EEG signals, such as the identification of facial expression and familiarity, as seen in the image. Previously, the effect of face inversion was investigated, and a significant and persistent delay of the N170 component was discovered. As a result, the types of emotional and familiarity stimuli were also separated according to the position of the face presented. Classification experiments were performed with reference models of EEG signals and a new improvement of the model was tested using the EEGNet SSVEP model with the regularity parameters L1 and L2. Due to the small data set, the data were augmented using the VAE model. The artificially generated signals in this way were used for pretraining, and the trained weights were reused in training on real signal data.

The EEG signal data are highly sensitive to subjects, and each subject produces different noise in the brain signals. EEG data are very sensitive to subjects, each person causing different noise in brain signals. However, due to the small data set, mixed training data from the seven older women were still used. Meanwhile, data from the oldest woman and data from a woman with AD were selected for testing to test the generalizability of the model. The relevance, strength, and valence of emotional input, as well as the impacted component of motor control of the face, are crucial considerations, according to Battalia et al. [68]. All of this evidence suggests that understanding how emotion is integrated into key executive functions such as inhibitory control is crucial not only for cognitive neuroscience but also for improving neurocognitive models of psychopathology. The inclusion of a patient with facial recognition impairment was to find out if cognitive and emotion detection in the brain signal was still encoded in the same manner as in a healthy individual. The visual processing parts of the brain remain unaffected. This would mean that parts of the visual processing of the brain of an Alzheimer’s patient are not affected by the disease. The findings of Borgomaneri et al. back up the idea that emotion perception prepares the body for action by highlighting the role of the right hemisphere in implementing a quick and brief facilitatory response to emotionally stimulating inputs such as emotional facial expressions [69].

## 7. Conclusions

The following conclusions were reached based on the results of the experiments:

The types of emotion and facial inversion stimulus were classified with the highest validation accuracy of 50.2% using the pretrained EEGNet SSVEP with the regularization model. Meanwhile, the data of the oldest person in the control group were correctly classified with 32.75% accuracy, and the Alzheimer’s patient data with 24.41% accuracy, which is poorer than a random classifier. The generalization abilities of the model are not great, considering the 17.5% gap in accuracy. On the other hand, it was observed that the trained model was guessing between emotion categories. There was also some evidence that the model was aware of the inversion effect in the data from the control group subject. The model does not reveal patterns in differentiating the position of the face or the type of emotion in a patient with AD.

The same CNN model outperformed the others in terms of familiarity and face-inversion stimuli. However, the model had a harder time distinguishing cognitive from emotional features. The accuracy values of the validation were 43.25% and 30.23% in the control group, and 27.72% in the Alzheimer’s patient data. With the investigation of confusion matrices, it was found that the least frequent cases of confusion are in the types of upright and inverted stimuli. This again demonstrates the model’s ability to learn the face-inversion effect. The improved model, the EEGNet SSVEP with the regularization of L1 and L2 in the final layer, demonstrated that the model’s ability to train was on average 1% better. As in other studies, an increase of 2% was observed. The effect of using pretrained weights trained on artificially generated data was almost negligible. However, this strategy may work better with more distinct classes.

## 8. Limitations and Future Directions

Summarizing all the findings, the classifier was unable to discriminate between the types of emotion (angry vs. neutral), nor the familiarity aspect (famous vs. unknown). Despite this, the model started to recognize the impact of face inversion. Consequently, the performance of the models needs to be further improved. Alternatively, the face-inversion characteristic should be removed from the classification of emotion and familiarity aspects, so as not to interfere with the model’s learning of less evident facial traits.

## Figures and Tables

**Table 1:** Classification of visual stimuli in other articles.

Article	Method	Visual Stimulus Types	Accuracy
Yang et al. [5]	RNN with data augmentation by randomly averaging	contraction/expansion/rotation/translation	73.72%, 61.74% (without augmentation)
Mishra and Bhavsar [7]	Siamese network	Object/Digits/Characters	77.9%/76.2%/74.8%
Cudlenco et al. [13]	Gabor filtering with Ridge Regression	Flowers/Airplanes/Cars/Park/Seaside/Old town	66.76%
Bagchi and Bathula [14]	EEG-ConvTranformer network	Human face/other 11 classes	78.37%
Spampinato et al. [53]	Recurrent Neural Networks (RNN)	40 objects from ImageNet	83%
List et al. [47]	Multivariate pattern classification analysis	Inverted/upright face	Less than 65%
Gunawan et al. [50]	DenseNet	Valence/arousal emotion types	60.55%

**Table 2:** EEGNet standard architecture. C—number of channels; T—number of time points; $F_{1}$—number of temporal filters; $F_{2}$—number of pointwise filters; D—number of spatial filters; $F_{s}$—sampling rate; N—number of classes.

Layer	Type	Filters	Size	Pad	Activation	Options
	Input					input = (C, T)
1	Conv2D	$F_{1}$	(1, T/2)	same	none	
	Batch Normalization					
2	DepthwiseConv2D		(C, 1)	valid	ELU	bias = False, depth multiplier = D, depth wise constraint = max norm (1.)
	Batch Normalization					
	AveragePooling2D		(1, 4)	valid		
	Dropout					rate = 0.5
3	SeparableConv2D	$F_{2}$	(1, 16)	same	ELU	bias = False
	Batch Normalization					
	AveragePooling2D		(1, 8)	valid		
	Dropout					rate = 0.5
4	Flatten					
Classifier	Dense	**N**			SoftMax	kernel constraint = max norm (0.25)

**Table 3:** DeepConvNet standard architecture. C—number of channels; T—number of time points; TC—length of temporal convolution; N—number of classes.

Layer	Type	Filters	Size	Pad	Activation	Options
	Input					input = (C, T)
1	Conv2D	25	TC	valid	none	kernel constraint = max norm (2)
	Conv2D	25	(C, 1)	valid	ELU	kernel constraint = max norm (2)
	Batch Normalization					epsilon = 1e-05 momentum = 0.1
	MaxPooling2D		(1, 2)	valid		Strides = (1, 2)
	Dropout					
2	Conv2D	50	TC	valid	ELU	kernel constraint = max norm (2)
	Batch Normalization					epsilon = 1e-05 momentum = 0.1
	MaxPooling2D		(1, 2)	valid		strides = (1, 2)
	Dropout					rate = 0.5
3	Conv2D	100	TC	valid	ELU	kernel constraint = max norm (2)
	Batch Normalization					epsilon = 1e-05 momentum = 0.1
	MaxPooling2D		(1, 2)	valid		strides = (1, 2)
	Dropout					rate = 0.5
4	Conv2D	200	TC	valid	ELU	kernel constraint = max norm (2)
	Batch Normalization					epsilon = 1e-05 momentum = 0.1
	MaxPooling2D		(1, 2)	valid		strides = (1, 2)
	Dropout					rate = 0.5
5	Flatten					
Classifier	Dense	N			SoftMax	kernel constraint = max norm (0.5)

**Table 4:** Visual stimulus types.

Experiment 2		Experiment 3	
Neutral	Fearful	Famous	Not famous
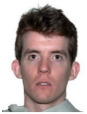	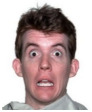	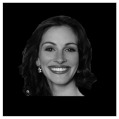	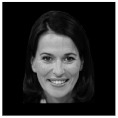
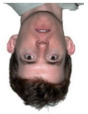	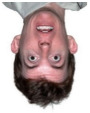	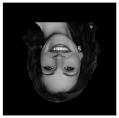	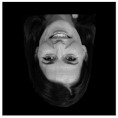

**Table 5:** VAE model training hyperparameters and model parameters.

Hyperparameter	Value	Model Parameter	Value
Epochs	1000	Kernel Size	5
Batch Size	32	Filters	16
Learning Rate	0.0001	Latent Dimension	2
Early Stopping Epochs	25	Optimizer	Adam

**Table 6:** VAE performance for each stimulus and class.

Dataset Name	Class Name	Training Time (s)	Validation Loss	Test Loss	Train Size	Val Size	Test Size
Emotion & view	fear/upright	1692.661	121.497	121.501	534	134	168
	fear/upsidedown	2405.592	114.304	116.231			
	neutral/upright	2442.686	114.250	115.465			
	neutral/upsidedown	2727.987	116.240	115.464			
Familiarity & view	famous/upright	2156.230	117.403	121.848	633	159	199
	famous/upsidedown	1541.902	116.805	121.415			
	unfamous/upright	2129.097	114.976	118.807			
	unfamous/upsidedown	3177.764	112.945	117.052			

**Table 7:** Values of training hyperparameters.

**Hyperparameter**	**Value**
Epochs	500
Batch Size	64
Learning Rate	0.001
Early Stopping Epochs	20
**Other settings**	**Value**
Optimizer	Adam

**Table 8:** Performance of models in training on artificially generated data.

Stimuli	Model	Run Time (s)	Test Acc	Test Loss	Val Acc	Val Loss	Number of Training Data	Number of Testing Data
**Familiarity & view**	DeepConvNet	82.79	1	0.001	1	0	1400	600
	EEGNet	203.39	0.985	0.055	0.994	0.053	1400	600
	EEGNet SSVEP	756.52	0.995	0.009	1	0.001	1400	600
	EEGNet SSVEP regularization	1523.41	1	0.017	1	0.015	1400	600
**Emotion & view**	DeepConvNet	38.59	1	0.002	0.938	0.129	1400	600
	EEGNet	134.89	1	0.026	0.998	0.028	1400	600
	EEGNet SSVEP	869.69	1	0.001	0.998	0.029	1400	600
	EEGNet SSVEP regularization	1304.23	1	0.015	1	0.016	1400	600

**Table 9:** The average training performance results with 5 repeated 2-fold cross validations of familiarity and view stimuli.

Model	Validation_Acc	Control_Acc	Alzheimer_Acc	Validation_Auc	Control_Auc	Alzheimer_Auc
DeepConvNet	0.378101	0.285379	0.275349	0.664231	0.530101	0.522043
EEGNet	0.416943	0.281388	0.272739	0.716867	0.526350	0.524505
EEGNet_SSVEP	0.428111	0.286462	0.263488	0.714708	0.543223	0.521810
EEGNet_SSVEP_regularization	0.434761	0.291155	0.279767	0.720801	0.551422	0.531294
augmented_DeepConvNet	0.414258	0.277798	0.271860	0.709143	0.533561	0.513521
augmented_EEGNet	0.435262	0.267870	0.273721	0.737287	0.513374	0.518509
augmented_EEGNet_SSVEP	0.439497	0.280686	0.279302	0.730503	0.536170	0.525493
augmented_EEGNet_SSVEP_regularization	0.432493	0.302347	0.277209	0.722147	0.552888	0.522122

**Table 10:** The average training performance results with 5repeated 2-fold cross validations of emotion and view stimuli.

Model	Validation_Acc	Control_Acc	Alzheimer_Acc	Validation_Auc	Control_Auc	Alzheimer_Auc
DeepConvNet	0.471126	0.317345	0.243897	0.793737	0.623122	0.499631
EEGNet	0.476327	0.312848	0.253756	0.809606	0.638286	0.510020
EEGNet.SSVEP	0.486617	0.332120	0.244131	0.802357	0.644521	0.503745
EEGNet_SSVEP_regularization	0.498247	0.329336	0.255634	0.806665	0.633405	0.508321
augmented_DeepConvNet	0.472622	0.314347	0.251643	0.793103	0.615264	0.492476
augmented_EEGNet	0.479862	0.311563	0.252582	0.812790	0.627414	0.512293
augmented_EEGNet_SSVEP	0.497408	0.315632	0.239437	0.807479	0.619471	0.496971
augmented_EEGNet_SSVEP_regularization	0.501958	0.327445	0.244131	0.804156	0.631282	0.501964

## Data Availability

The dataset used in this study is available from https://figshare.com/articles/dataset/Face_recognition_deficits_in_a_patient_with_Alzheimer_s_disease_amnesia_or_agnosia_/11913243/1 (accessed on 20 December 2021).
